# Bending for love: losses and gains of sexual dimorphisms are strictly correlated with changes in the mounting position of sepsid flies (Sepsidae: Diptera)

**DOI:** 10.1186/1471-2148-8-155

**Published:** 2008-05-21

**Authors:** Nalini Puniamoorthy, Kathy Feng-Yi Su, Rudolf Meier

**Affiliations:** 1 Department of Biological Sciences, National University of Singapore, 14 Science Dr 4, Singapore 117543, Singapore

## Abstract

**Background:**

Sexually dimorphic structures contribute the largest number of morphological differences between closely related insect species thus implying that these structures evolve fast and are involved in speciation. The current literature focuses on the selective forces that drive these changes, be it 'sexual conflict' or 'female choice'. However, there are only few studies examining the function of sexual dimorphisms and even fewer that investigate how functional changes influence dimorphisms. This is largely due to the paucity of taxa for which the morphology, behavior, and phylogenetic relationships for multiple species are known. Here we present such data for sepsid flies. Sepsids have starkly dimorphic forelegs whose function can be documented under laboratory conditions. We use data from 10 genes to reconstruct the phylogenetic relationships for 33 species and test whether mounting positions are correlated with the presence and absence of sexual dimorphisms in the forelegs.

**Results:**

The phylogenetic tree fully resolves the relationship with 29 of the 31 nodes of the tree having a posterior probability of 1.0. Twenty-eight of the 31 sepsid species have sexually dimorphic forelegs. All 28 species with such forelegs have the same mounting technique whereby the male uses his modified forelegs to grasp the female wingbase. Mapping mounting behavior and foreleg morphology onto the tree reveals that the wing grasp evolved once and was reduced twice. All changes in the mounting behavior are strictly and statistically significantly correlated with the origin and losses of sexually dimorphic legs (concentrated changes test: P < 0.001); i.e., the two species that have independently lost the wing grasp have both also re-evolved monomorphic legs. The wing grasp in these species is replaced with a novel but very similar mounting technique not involving the forelegs: the males bend their abdomens forward and directly establish genital contact to the female. In addition, one of the secondarily monomorphic species, *Sepsis secunda*, has evolved a new sexual dimorphism, a 'bump' on the dorsal side of the 4th tergite, which is now touching the ventral side of the female abdomen.

**Conclusion:**

Our study reveals that the evolution of sexually dimorphic legs in Sepsidae can only be understood once the function of the legs during mating is considered and the relationships of species with and without sexual dimorphisms are known. We demonstrate that homoplasy in sexually dimorphic structures can be due to homoplasy in mating behavior. We furthermore document that the two species with secondarily monomorphic legs have independently replaced the typical sepsid wing grasp with very similar, new mounting techniques. This suggests that convergent evolution may be common in mating behaviors.

## Background

Most closely related species of insects differ exclusively or predominantly with regard to sexually dimorphic traits [1,2]. This observation implies that these traits evolve fast and are likely to be involved in speciation. Understanding the evolution of sexual dimorphisms is thus crucial for understanding the impressive species diversity of the Insecta, the most speciose clade of multicellular animals [3]. This has made the study of sexual dimorphisms also one of the most important goals of modern evolutionary biology.

Much of the literature focuses on the theoretical issues that drive the sexual selection that is responsible for the origin and maintenance of sexual dimorphisms. Traditionally, the fast evolution of male ornaments had been attributed to selection via female choice [4]. Some empirical data and genetic models had shown that this choice could be driven by direct benefits that increase survivorship [5,6] or indirect genetic benefits that could improve viability and/or the attractiveness of the offspring [7-12]. However, there is an alternative explanation for the fast evolution of sexual dimorphisms. The unequal investments in gametes by males and females leads to a conflict of interest between the sexes ("sexual conflict" [13,14]). Males have a higher interest in multiple matings than females and may thus try to coerce females into being polygamous. As a result both sexes can become involved in a sexually antagonistic arms race and evolve mechanisms to gain control over reproduction, regardless of the costs to the other sex [15,16]. Evidence for such morphological coevolution between male and female reproductive characters has been found across a number of insect groups like Coleoptera (e.g.: Bruchidae and Dytiscidae), Hemiptera (e.g.: Gerridae) and Diptera (e.g.: Empididae and Scathophagidae) [17-25].

However, comparatively little is known about the function of many sexually dimorphic structures and even less is known about how the function of these structures changes over evolutionary time. One reason for this gap in our knowledge is that studying the evolution of function requires concurrent information on the morphology, behavior and phylogeny of multiple species. Only then can functional changes be mapped onto a phylogenetic hypothesis. Unfortunately, for many sexually dimorphic features, only the morphology has been documented in detail and it is often unclear how they are being used. This is particularly so for male genitalia whose function is difficult to study because they interact with internal elements of the female reproductive tract [26,27]. Even if the function for a particular structure has been studied in great detail, information is usually only available for one or a few species [28] and if there is information for several species, the phylogenetic relationships are frequently unknown. As a consequence there are only few studies that have documented the evolution of the function of sexually dimorphic structures [18,29-33]. Here, we use a wealth of information on the morphology and function of the male forelegs for 31 species of Sepsidae (Diptera: Cyclorrhapha) to document how sexual dimorphisms are correlated with mating behavior over evolutionary time.

**Figure 1:**
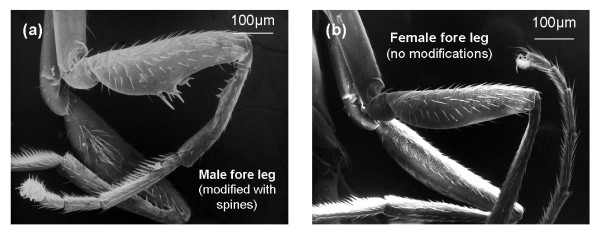
**Forelegs of *Sepsis dissimilis *Brunetti, 1910**. (a) Male foreleg; (b) Female foreleg.

**Figure 2:**
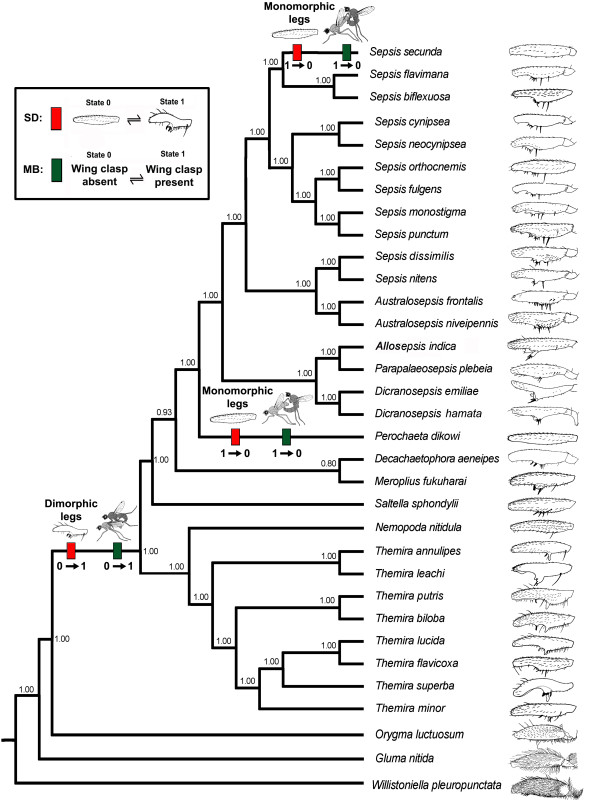
**Evolution of sexual dimorphism (SD) and mounting behavior (MB) in Sepsidae**. Red hatchmarks represent sexually dimorphic forelegs and green hatchmarks represent the mounting behavior. Numbers below hatchmarks indicate character state number. Posterior probability values are indicated at the branch nodes.

Over the past 30 years, sepsid flies have become model organisms in the study of behavior in general and sexual selection in particular [34-43]. Sepsids are ideal models because some of the structures involved in the interactions between males and females are easily accessible and display stark sexual dimorphism [44,45]. Furthermore, sepsids can be maintained in the laboratory and some aspects of their mounting and copulation behavior can be video-taped and studied without dissection. The most interesting sexually dimorphic structure that is easily accessible is the forelegs. In most sepsid species, the males use their forelegs for grasping the female wing base prior to copulation. The forelegs are often strongly sexually dimorphic, with female legs being unmodified while the male legs display a wide variety and species-specific cuticular outgrowths, indentations, and/or modified bristles (see Figures 1 and 2). The males of most sepsid species will indiscriminately jump on females and attempt to clamp to the female's wing base. It is here that the morphological modifications of the male forelegs play a major role. They tightly mesh with the wing veins and cells of the female wing [44,45] and help the male during the next stage of mating, a rodeo-like struggle that is found in most species and that starts after the male has mounted the female [34,46-48]. The females of many sepsid species use vigorous shaking in order to dislodge and/or test the stamina of the male [40,43,49,50]. In addition, females usually bend their abdomen ventrally so that the males cannot establish genitalic contact without female consent [34,42]. Copulation can only be initiated once the female lifts the tip of her abdomen, which may or may not occur in response to male behaviors [38,51,52]. Many copulation attempts by males are ultimately aborted either because the males voluntarily dismount or because males are dislodged through shaking [38].

Sexually dimorphic forelegs are found in the vast majority of sepsid species. However, monomorphic legs are known for six species: *Orygma luctuosum *Meigen 1830, three species of *Perochaeta *Duda 1926, *Sepsis duplicata *Haliday 1838, and *S. secunda *(Melander et Spuler 1917). Here, we document the foreleg morphology and mounting behavior for two outgroup species, 3 species of Sepsidae with monomorphic legs and 28 species of Sepsidae with sexually dimorphic legs. We reconstruct the phylogenetic relationships among all species based on sequence data for 10 genes, map foreleg morphology and mounting behavior onto the phylogenetic tree, and use a concentrated change test [53] to establish whether the gains and losses of dimorphic forelegs are correlated with changes in how the forelegs are used during mounting.

## Methods

### Morphology

The forelegs of ethanol-preserved males and females for the 33 species were removed from the thorax and both the anterior and posterior views of the femur, tibia and trochanter were documented at 200× and 400× magnification using a Hirox microscope and the software Digi-Scale. Drawings of the anterior view of the male femur and trochanter were prepared based on these digital images and comparisons with additional specimens of the same species. The morphology of the male foreleg was then compared to the morphology of the female foreleg. Legs were regarded as sexually monomorphic when male and female forelegs could not be differentiated from each other upon removal from the thorax. For certain select species, a Jeol JSM-7220A Scanning Electron Microscope was used to document additional dimorphic structures.

### Rearing and Mating experiments

Laboratory cultures were established for 30 sepsid species based on females collected in the field. The parental cultures were maintained under controlled conditions in incubators and supplied regularly with dung and sugar water. Mating experiments involved virgin flies that were obtained by sexing newly emerged flies within 24 hours of eclosion, keeping males and females as virgins in separate containers. Mating trials were carried out approximately four days after separation. A male and a female were introduced into a small Petri dish and their interaction was recorded using a Leica MZ16A Microscope camera and a VCR. The recording started upon the introduction of both flies into the Petri dish and ended after a successful copulation or after 25 minutes if the males did not attempt to mount. The analog recordings were digitized using the non-linear editing software, Final Cut Pro, and studied frame by frame (1 frame = 1/25 seconds) in order to document the mounting behavior of the different species in detail. The number of recordings per species is listed in the results section. Information on the mating behavior of *Saltella sphondylii *was obtained from an earlier study by Martin and Hosken [54].

### Phylogenetic Relationships

We reconstructed the relationships among the 33 species based on 10 genes (18S, 28S, AATS, EF1a, Histone 3, 12S, 16S, COI, COII, CYB; for details see [55]; for Genbank accession numbers [see Additional file 1]). The data set was subjected to a Bayesian analysis as implemented in MrBayes 3.1 [56]. We used MrModeltest [57] to establish that the GTR + I + G model was favored for all genes by both the Akaike information criterion and hierarchical likelihood ratio testing. The data set was analyzed for 3,000,000 generations and a tree was sampled every 300 generations (=10,000 trees) with a subsequent burn-in of 2500 trees.

### Character mapping and concentrated change test

Sexual dimorphism (SD) was scored as a presence/absence character (sexually dimorphic legs absent = 0; present = 1). With regard to the mounting behavior (MB), we used the role of the forelegs to define two character states. State 0 was used to code those species where the male foreleg is not used to grasp the female wing base. Presence of the grasp was coded as character state 1. To test whether changes in behavior and morphology are correlated, we mapped both binary characters onto the phylogenetic tree and used the concentrated change test [53] as implemented in MacClade 4 [58] using the Sepsidae node as the target clade.

## Results

### Morphology

Out of the 31 sepsid species included in this study, the following 28 have sexually dimorphic forelegs. The number of mating trials with successful mountings is listed after the species names: *Australosepsis frontalis *(Walker, 1860), n = 15; *A. niveipennis *(Becker, 1903), n = 17; *Decachaetophora aeneipes *(de Meijere, 1913), n = 10; *Dicranosepsis emiliae *(Ozerov, 1992), n = 13; *D. hamata *(de Meijere, 1911), n = 9; *Meroplius fukuharai *(Iwasa, 1984), n = 5; *Nemopoda nitidula *(Fallen, 1820), n = 8; *Parapaleosepsis plebeia *Duda, 1926, n = 15; *Saltella sphondylii *(Schrank, 1803), *Sepsis biflexuosa *Strobl, 1893, n = 3; *S. cynipsea *(Linnaeus, 1758), n = 20;*S. dissimilis *Brunetti, 1910, n = 20; *S. flavimana *Meigen, 1826, n = 5; *S. fulgens *Meigen, 1826, n = 10;*S. (Allosepsis) indica *Wiedemann, 1824, n = 20; *S. monostigma *Thomson, 1869, n = 5; *S. neocynipsea *Melander et Spuler, 1917, n = 5;*S. nitens *Wiedemann, 1824, n = 7; *S. orthocnemis *Frey, 1908, n = 6;*S. punctum *(Fabricius, 1794), n = 10; *Themira annulipes *(Meigen, 1826), n = 4; *T. biloba *Andersson, 1975, n = 3; *T. flavicoxa *Melander et Spuler, 1917, n = 10;*T. leachi *(Meigen, 1826), n = 3;*T. lucida *(Staeger, 1844), n = 15;*T. minor *(Haliday, 1833), n = 15;*T. putris *(Linnaeus, 1758) n = 15; and *T. superba *(Haliday, 1833) n = 20. The forelegs of *N. nitidula *may at first sight appear almost monomorphic, but this is due to the fact that the dimorphic elements are very small. They are thus here documented in a separate micrograph [see Additional file 2]. The three species with sexually monomorphic forelegs in our study were *Orygma luctuosum*, n = 14, *Perochaeta dikowi *Ang et al., 2008, n = 20 and *Sepsis secunda*, n = 8. The two outgroups, *Gluma nitida *McAlpine, 1991 and *Willistoniella pleuropunctata *(Wiedemann, 1824) also have monomorphic forelegs (see Figure 2).

### Mounting behavior

**Figure 3:**
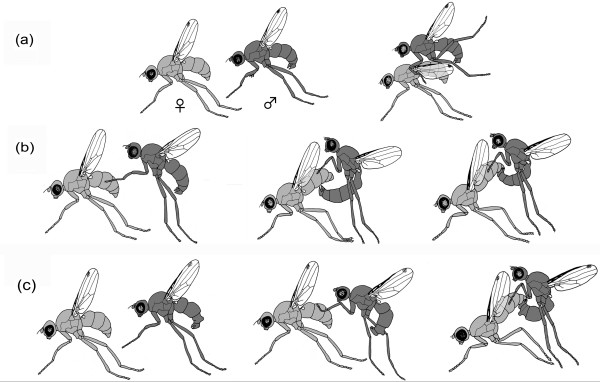
**Mounting techniques in Sepsidae**. (a) "Typical" sepsid mount: male uses modified forelegs to clasp female wingbase; (b) Novel mounting technique in *Perochaeta dikowi*: male bends abdomen ventro-anteriad and makes contact with the ventral side of the female abdomen with his surstylus before sliding posterior to establish genital contact; (c) Novel mounting technique in *Sepsis secunda*: male bends abdomen ventro-anteriad to establish direct genital contact.

The mounting behavior was documented for all of the above mentioned sepsid species except *Saltella sphondylii *for which the behavior has been described in the literature [54]. All 28 species with sexually dimorphic legs exhibit the "typical" sepsid mount where the male jumps/climbs onto the female and uses his modified forelegs to clasp onto the base of her wing (see Figure 3a). Video clips documenting the mounting behavior are provided as additional files [see Additional files 3, 4 and 5] (or visit the following websites [59-61]). However, the three species that have monomorphic forelegs have mounting techniques not involving the male foreleg. The males of *Orygma luctuosum *jump on the female and wrap their fore and mid legs around the female thorax [see Additional file 6] (or visit the following website [62]). This is followed by a brief struggle whereby the female either attempts to shake off the male, often using her legs to 'kick' him, or by rolling over in order to dislodge the male. In successful mating trials, upon establishing genital contact, the male rests his forelegs on the female's postpronotal callus and not around the wing base.

The mounting behaviors of the two other species with monomorphic legs, *Sepsis secunda *and *Perochaeta dikowi*, are very similar to each other but differ considerably from the behavior of *O. luctuosum*. In *P. dikowi *(see Figure 3b) and *S. secunda *(see Figure 3c) males approach the females from behind and bend their abdomen ventro-anteriad in order to directly establish genital contact without the use of the male forelegs. Once genital contact has been established, copulation starts. The only difference between the two species is that in *P. dikowi*, the males initially extend their surstylus beyond the female genitalia and touch the ventral side of the female abdomen near sternite three. The surstylus then slides posterior before establishing genital contact [see Additional file 7] (or visit the following website [63]). During copulation the male forelegs rest on the females' postpronotal callus (for a detailed description of mating behavior see [64]). In *S. secunda *the males will first establish genital contact and then place their forelegs loosely and mid-way along the anterior edge of the female wing [see Additional file 8] (or visit the following website [65]).

### Phylogeny and character evolution

The dataset was subjected to a Bayesian analysis and three independent analyses resulted in similar tree topologies, comparable clade probabilities and substitution model parameters. This suggested that consistent estimates of the posterior probability (PP) distributions had been obtained. Our analysis yielded a very well resolved tree placing *Orygma luctuosum *as sister group to all the remaining sepsids. All but two nodes had PP values of 1.00 (see Figure 2). The dimorphism and behavior characters were mapped onto the phylogenetic hypothesis. The presence of a sexually dimorphic foreleg is strictly correlated with a mating position involving the wing grasp (concentrated changes test using all of Sepsidae as target clade: two losses in SD with two losses in MB, *P *= 0.000742). All three changes in the sexual dimorphism character occur on the same branches as the changes in mounting behavior (see Figure 2).

## Discussion

**Figure 4:**
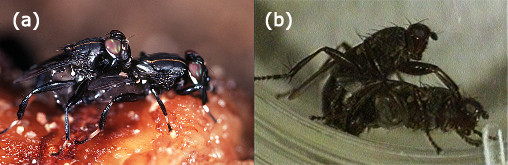
**Flies in copula**. Male forelegs not clasping female wingbase. (a) *Willistoniella pleuropunctata*; (b) *Orygma luctuosum*.

Mounting behavior in Sepsidae is strictly and statistically significantly correlated with the origin and losses of sexual dimorphisms. The "typical" mounting position of Sepsidae involving the wing grasp only evolved once and within the family (see Figure 2; red hatchmark) and the origin of this mounting position coincides with the origin of sexually dimorphic forelegs (see Figure 2; green hatchmark). The males of the three sepsid species with monomorphic forelegs, *Orygma luctuosum*, *Perochaeta dikowi*, and *Sepsis secunda *place the forelegs on different parts of the female body. Given its placement on the tree, the mounting behavior and lack of sexual dimorphism is plesiomorphic in *O. luctuosum*. In fact, not only do the forelegs of *O. luctuosum *closely resemble the legs of the outgroups (see Figure 2) but the mounting behavior is also very similar to the behaviors of the outgroups *Gluma nitida *[66] and *Willistoniella pleuropunctata *(see Figure 4). For instance, male coelopids have been observed to mount females and wrap their legs around the female thorax and as observed in *O. luctuosum*, the females will 'kick' the males with their midlegs and even roll over [67,68]. Other acalyptrate flies like *Protopiophila litigata *(Diptera: Piophilidae) and *Dryomyza anilis *(Diptera: Dryomyzidae) also exhibit similar mounting behaviors. For example, upon mounting, *P. litigata *males rest their fore tarsi on the female postpronotum [69] while in *D. anilis*, the male forelegs are placed on the female head [70].

However, the monomorphic legs of *Perochaeta dikowi *and *Sepsis secunda *are due to the convergent loss of the male leg armature in two distantly related clades of Sepsidae. A study of the mating behavior reveals that in both species, the forelegs are no longer used for grasping the female wingbase. One can imagine many different ways of replacing the typical sepsid mounting position with a new mounting technique. Yet, *P. dikowi *and *S. secunda *have convergently invented near identical, new behaviors. In both species, the male approaches the female from behind. In both species, the males curl their abdomens forward in order to touch the female abdomen with their claspers. In both species, it is only afterwards that the male legs play a minor role in mating: in *P. dikowi*, males rest their fore tarsi on the female postpronotal callus whilst the males of *S. secunda *rest their fore tarsi mid-way along the female wing margin. Convergence in morphology is often driven by similarities in habitat and environment. However, there are no aspects of the known autecology of these two species that could explain the convergent evolution of the mounting behavior. Both are typical scavenger flies that use cow dung as breeding substrate. *Sepsis secunda *is mostly found on open pastures in hot and dry areas of North America, while *P. dikowi *is only known from forests at mid-elevation sites in the tropics [64]. Furthermore, both species co-occur in the same habitat with other sepsid species that have the "normal" wing grasp.

Evolutionary biologists have long predicted and documented convergence in sexually dimorphic structures [45]. Some authors attribute the convergent evolution of correlated traits to highly conserved patterns of pleiotropy as a potential mechanism for channeling sexual shape dimorphism in Diptera [71]. These hypotheses address the proximate causes for the convergent evolution of sexual dimorphisms and should be complemented with studies that can address the ultimate causation. We suggest that the ultimate reasons for why similar genetic architecture may be utilized to produce convergent dimorphisms are convergent changes in mating behavior. In our case the convergent loss of the wing clamp leads to the convergent loss of the leg dimorphisms. Yet, more intriguing is our finding of convergent evolution of a very specific new mounting behavior which may suggest that highly conserved patterns of pleiotropy and a conserved genetic architecture may also exist for behavior.

We had previously outlined that there is only scant information on how the function of sexually dimorphic traits change over time. Our study helps to fill the void. In the sepsid outgroups and *Orygma luctuosum *the fore femora and tibiae of the males function like the same structure in most flies. However, in most species of Sepsidae the male forelegs acquire an additional function, i.e., the clamping of the females' wingbase and as a result a new sexual dimorphism evolves. Subsequently, the male forelegs undergo extensive and fast modification as revealed in Figure 1 where all 28 species with sexually dimorphic legs have species-specific modifications. However, as *P. dikowi *and *S. secunda *document sexual dimorphisms can also disappear once a structure loses its role in mating behavior.

There are still relatively few studies that document how a behavioral change is correlated with a morphological change [18,29,31,32]. However, a similar case is also known from seaweed flies (Diptera: Coelopidae) where the male foretarsus touches the female antenna during mating [72]. A new morphological structure, a thumbnail-like process, evolved on the basitarsus of males and it must be evolving rapidly because it differs between the different species of Coelopidae [73]. The antlered flies in the genus *Phytalmia *(Diptera: Tephritidae) provide another example. Males have cuticular projections on the head (antlers) that are used in male-male combat for territory and the complexity of the antler shapes differs quite drastically between species [74]. Species with complex antlers have simple behavioral repertoires whilst those with simpler antlers evolved more complex behaviors involving the fore, mid and hind legs (e.g.: 'wing-locking' and 'stilting' [33]).

**Figure 5:**
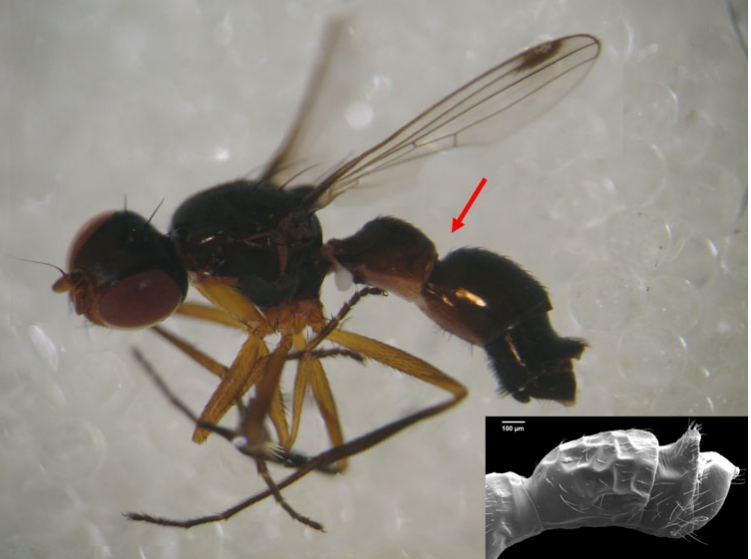
***Sepsis secunda***. Habitus picture of male; SEM of sexually dimorphic dorsal 'bump'.

The novel mounting technique in *Perochaeta dikowi *and *Sepsis secunda *is not only associated with the loss of sexually dimorphic legs, but also associated with the gain of new sexual dimorphisms. In *P. dikowi *the males have modified fourth or fifth sternites that form brushes. These are used during copulation [64]. In *S. secunda*, the males have sexually dimorphic 4^th ^tergites. Normally, this tergite is monomorphic which is not surprising given that the dorsal side of the male abdomen is not involved in the mating behavior of most sepsids. However, in *S. secunda *the male tergites touch the female when the male abdomen is curled forward [see Additional file 8] (or visit the following website [65]). A new dorsal, bump-like extension of the tergite four evolves and this tergite becomes strongly sexually dimorphic (see Figure 5; SEM picture). In addition, *S. secunda *has an elongated abdomen (elongated segments I & II). In particular, the posterior part of the segment II is strongly lengthened and connected to a distinctly constricted tergite three (see Figure 5; red arrow). This constriction is likely related to the new mating behavior that requires the male to bend his abdomen forward. Indeed, this constriction closely resembles the wasp waist of parasitoid wasps that need to have similar flexibility in order to use their ovipositor for injecting poison and/or eggs into a host. It appears likely that these morphological changes in *S. secunda *occurred rapidly because they are not found in its sister group, *S. duplicata*, and the two species are very similar with regard to the barcoding gene COI (1.97%; normal distances in most closely related Diptera species > 3% [75]).

Throughout the discussion, we often implicitly assumed that the change in behavior preceded the loss of the sexual dimorphism although technically the strict correlation between the two traits could also imply the reverse order. However, based on functional considerations it appears unlikely that the reduction of the male leg armature preceded the behavioral change given that the leg armature has been experimentally shown to be important for successful mating in sepsids [37,44,51,76]. Reversing the order of events would also not alter our finding of a strict correlation between mounting behavior and the losses and gains of sexual dimorphisms in Sepsidae.

Several groups of Diptera are important models for investigating sexual conflict. A number of studies have documented coevolution between male and female reproductive characters. For instance, Minder *et al. *(2005) show that the sperm length in males evolves with the length of spermathecal ducts in females across several species of yellow dung flies (Scathophagidae) [22]. In *Drosophila melanogaster *(Drosophilidae) female resistance evolves in response to the harm caused by male accessory gland proteins that reduce female fitness and are transferred during copulation [77-80]. Furthermore, Bonduriansky and Rowe (2005) argued that intralocus sexual conflict reduces the heritability of sexual traits in *Prochyliza xanthostorna *(Piophilidae) and Otronen (1994) suggested that inter-sexual conflict over mating patterns led to repeated copulations in *Dryomyza anilis *(Dryomyzidae) [81]. In Sepsidae, some authors argued for sexual conflict [38,47,82] while others claimed that the evolution of sexual dimorphisms is driven by female choice [35,37].

In our study, we have identified several elements that are characteristic of an arms race: morphological variation among close relatives as well as a correlation between morphology and behavior. However, we only documented this coevolution within the male sex and in order to document sexual conflict, we would also need to document coevolutionary responses in the females (see [45]). In the absence of this evidence, we cannot attribute the diversity of male foreleg armature to sexually antagonistic coevolution.

## Conclusion

One of the most powerful indirect techniques in evolutionary biology is using the comparative method to investigate convergent evolution [83]. By combining behavioral, morphological, and phylogenetic data, we are here able to show that sexual dimorphisms evolve fast and convergently in Sepsidae. Homoplasy is not restricted to sexual dimorphisms, but also affects mounting behavior. Our study furthermore documents how important it is to study the function of sexually dimorphic structures.

## Authors' contributions

NP conducted all the mating behavior experiments in this paper. Both NP and KF–YS were responsible for the morphological documentation of the various sexually dimorphic and monomorphic structures. The molecular work i.e. DNA matrix assembly was primarily carried out by KF–YS with help from Ms. Sujatha N. Kutty. RM contributed to the conception of this research and performed all the phylogenetic analyses in the paper. All authors read and approved the final manuscript.

## Supplementary Material

Additional file 1 Table 1. List of species and Genbank accession numbers for 10 genes. Click here for file

Additional file 2 Figure 6 (Male foreleg of *Nemopoda nitidula*). Weakly modified male foreleg with an anterior bristle and short row of posterior spines as indicated by red arrows. Click here for file

Additional file 3 Video A1 (Themira + Nemopoda). Compiled video of mounting clips and foreleg drawings for eight Themira species and Nemopoda nitidula, all with sexually dimorphic forelegs. Click here for file

Additional file 4 Video A2 (Australosepsis + Sepsis). Compiled video of mounting clips and foreleg drawings for two Australosepsis species and 10 Sepsis spp., all with sexually dimorphic forelegs. Click here for file

Additional file 5 Video A3 (Five genera of sepsid species). Compiled video of mounting clips and foreleg drawings for Decachaetaphora aeneipes, Meroplius fukuharai, Allosepsis indica, Parapaleosepsis plebeia, and two Dicranosepsis species, all with sexually dimorphic forelegs. Click here for file

Additional file 6 Video B1 (Orygma luctuosum). Video of mounting clips and foreleg drawing for Orygma luctuosum with sexually monomorphic forelegs. Click here for file

Additional file 7 Video B2 (Perochaeta dikowi). Video of mounting clips and foreleg drawing for Perochaeta dikowi with sexually monomorphic forelegs. Click here for file

Additional file 8 Video B3 (Sepsis secunda). Video of mounting clips and foreleg drawing for Sepsis secunda with sexually monomorphic forelegs. Click here for file
