# Supplementary figures and images for: Bending for love: losses and gains of sexual dimorphisms are strictly correlated with changes in the mounting position of sepsid flies (Sepsidae: Diptera)
Source: BMC Evol Biol. 2008 May 21;8:155. doi: 10.1186/1471-2148-8-155 (PMC2409323; doi:10.1186/1471-2148-8-155)

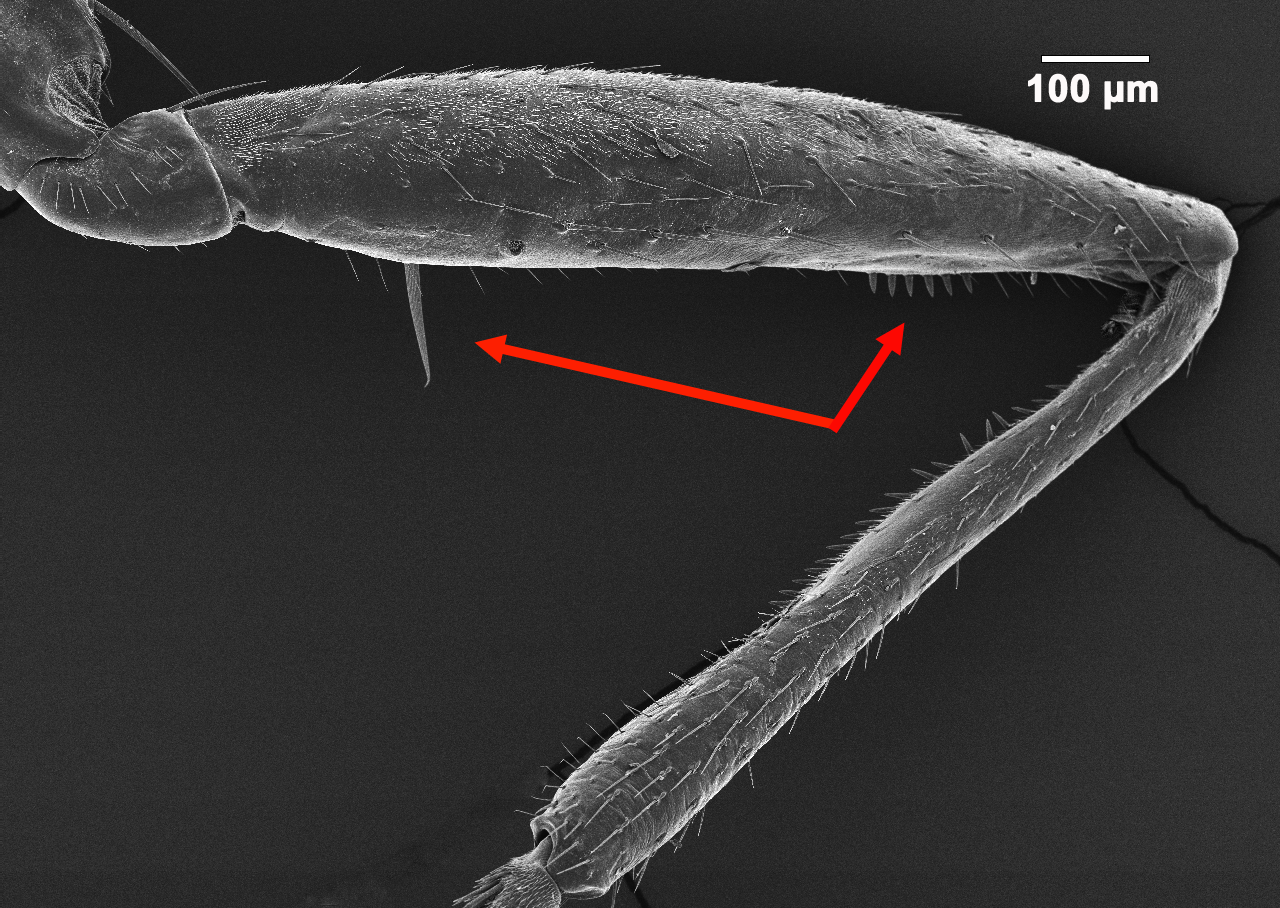

Supplement: Additional file 2 — Figure 6 (Male foreleg of Nemopoda nitidula). Weakly modified male foreleg with an anterior bristle and short row of posterior spines as indicated by red arrows. [file 1471-2148-8-155-S2.png]
